# Cubic, hexagonal and tetragonal FeGe_*x*_ phases (*x* = 1, 1.5, 2): Raman spectroscopy and magnetic properties†

**DOI:** 10.1039/d1ce00970b

**Published:** 2021-08-20

**Authors:** A. Kúkoľová, M. Dimitrievska, A. P. Litvinchuk, S. P. Ramanandan, N. Tappy, H. Menon, M. Borg, D. Grundler, A. Fontcuberta i Morral

**Affiliations:** Laboratory of Semiconductor Materials, Institute of Materials, School of Engineering, Ecole Polytechnique Fédérale de Lausanne (EPFL) 1015 Lausanne Switzerland mirjana.dimitrievska@epfl.ch anna.fontcuberta-morral@epfl.ch; Laboratory of Nanoscale Magnetic Materials and Magnonics, Institute of Materials, Ecole Polytechnique Fédérale de Lausanne (EPFL) 1015 Lausanne Switzerland; Texas Center for Superconductivity at UH, Department of Physics, University of Houston USA; Electrical and Information Technology, Lund University Lund Sweden; NanoLund, Lund University Lund Sweden; Institute of Electrical and Micro Engineering, School of Engineering, Ecole Polytechnique Fédérale de Lausanne (EPFL) 1015 Lausanne Switzerland; Institute of Physics, School of Basic Sciences, Ecole Polytechnique Fédérale de Lausanne (EPFL) 1015 Lausanne Switzerland

## Abstract

There is currently an emerging drive towards computational materials design and fabrication of predicted novel materials. One of the keys to developing appropriate fabrication methods is determination of the composition and phase. Here we explore the FeGe system and establish reference Raman signatures for the distinction between FeGe hexagonal and cubic structures, as well as FeGe_2_ and Fe_2_Ge_3_ phases. The experimental results are substantiated by first principles lattice dynamics calculations as well as by complementary structural characterization such as transmission electron microscopy and X-ray diffraction, along with magnetic measurements.

## Abstract 2

Structural properties of various FeGe_*x*_ compounds are explored by Raman spectroscopy in order to provide reference Raman spectra, and enable fast and local phase determination in FeGe thin films.

## Graphical abstract

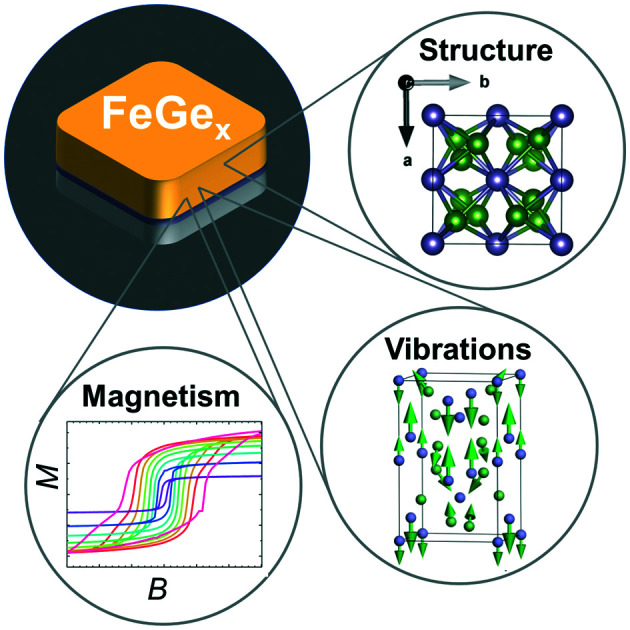

## 1 Introduction

Fe–Ge compounds have been attracting interest due to their enticing magnetic and thermoelectric properties. Thanks to the particular crystal symmetry of the cubic B20 phase, FeGe can host topologically protected spin structures such as skyrmions. As a consequence, B20 FeGe structures exhibit great potential as building blocks for logic or data storage devices.^1–7^ On the one hand, the hexagonal (B35) FeGe phase is a uniaxial antiferromagnet along the *c* axis.^8^ On the other hand, the monoclinic FeGe phase possesses a complex magnetic structure with an overall antiferromagnetic behaviour.^9^ Most of the iron-rich compounds with a hexagonal (B8_2_) β or η-Fe_*x*_Ge (1.32 < *x* < 1.67) structure exhibit a ferromagnetic nature.^10–12^ Apart from its interesting magnetic properties,^13,14^ Fe_2_Ge_3_ has been studied for its high thermoelectric performance.^15^ In addition, FeGe nanoparticles confined in an iron oxide matrix have shown storage potential in Li-ion batteries.^16^

The fabrication methods for FeGe thin films reported in the literature are molecular beam epitaxy (MBE), sputter beam epitaxy and magnetron sputtering on Si, Ge and MgO substrates. The growth on Si(111) led to the formation of epitaxial B20 thin films exhibiting a 0.05% lattice mismatch when grown by MBE^17^ and a compressive strain of 0.32% along the (111) direction (out-of-plane) and a tensile strain of 0.8% in the plane when co-sputtered under ultrahigh vacuum (UHV).^18^ The sputtering on Si(111) was shown to lead to phase-pure epitaxial B20 films^3,19^ with less than 0.5% impurity phases. The magnetron sputtering of FeGe on MgO(001) substrates led to B20 thin films with a compressive strain of 1.1%.^20^ Phase-pure epitaxial B20 FeGe films grown on a Ge(111) substrate through sputter beam epitaxy exhibited ∼4% tensile strain.^21^ Single crystal growth on Ge(001) with various Fe compositions using an electron beam evaporator in a UHV chamber revealed the coexistence of monoclinic and hexagonal phases of Fe_*x*_Ge for 1 < *x* < 1.2 due to the small lattice mismatch with the substrate regardless of the growth temperature or time.^12^

While X-ray diffraction (XRD) patterns have been established for the majority of Fe–Ge phases, their vibrational characterization has been limited. Raman spectroscopy, besides being a widely used tool for structural characterization, may also serve as a powerful technique for phase identification at the micro-scale, with shorter acquisition times when compared to other techniques.^48^ This is a major advantage over XRD characterization, especially in cases where probed phases have similar structures, leading to the overlap of XRD peaks and rendering phase identification difficult.^49^

The phonon structure of Fe–Ge compounds has not been widely explored. Vibrational modes have been theoretically studied for an FeGe (B20) monolayer by Bhuyan *et al.*,^22^ where lattice dynamics calculations have identified phonon modes at 102, 127, 147, 240 and 280 cm^−1^. Theoretical and experimental studies on other cubic B20 structures such as MnSi (also a chiral magnet^23^), FeSi, CoSi, and RuSi have reported similar phonon modes with the most prominent Raman peaks at 190 and 310 cm^−1^.^24,25^ However, detailed studies on the vibrational properties of many FeGe phases are still missing.

In this work we perform detailed structural and vibrational characterization of cubic, hexagonal and tetragonal FeGe_*x*_ phases (*x* = 1, 1.5, 2). Lattice dynamics calculations based on density functional theory (DFT) in correlation with Raman measurements have been performed in order to provide reference Raman mode positions of these phases and enable their easier identification. Fe–Ge thin films with [Fe]/([Fe] + [Ge]) compositions in the range from 0.35 to 0.50 have been prepared. Structural characterization and initial phase identification have been done using XRD in various geometries, such as grazing incidence X-ray diffraction (GIXRD), wide angle grazing incidence X-ray scattering (GIWAXS) and Bragg–Brentano geometry (*θ*–2*θ*) measurements. This has enabled differentiation of phases present in the surface and bulk of the films. These results were supported by morphological and compositional assessment performed by transmission electron microscopy (TEM) and energy dispersive X-ray (EDX) analysis. Finally, the magnetization of thin films has been measured and discussed in terms of the phases present.

## 2 Phase diagram of Fe–Ge

**Fig. 1:**
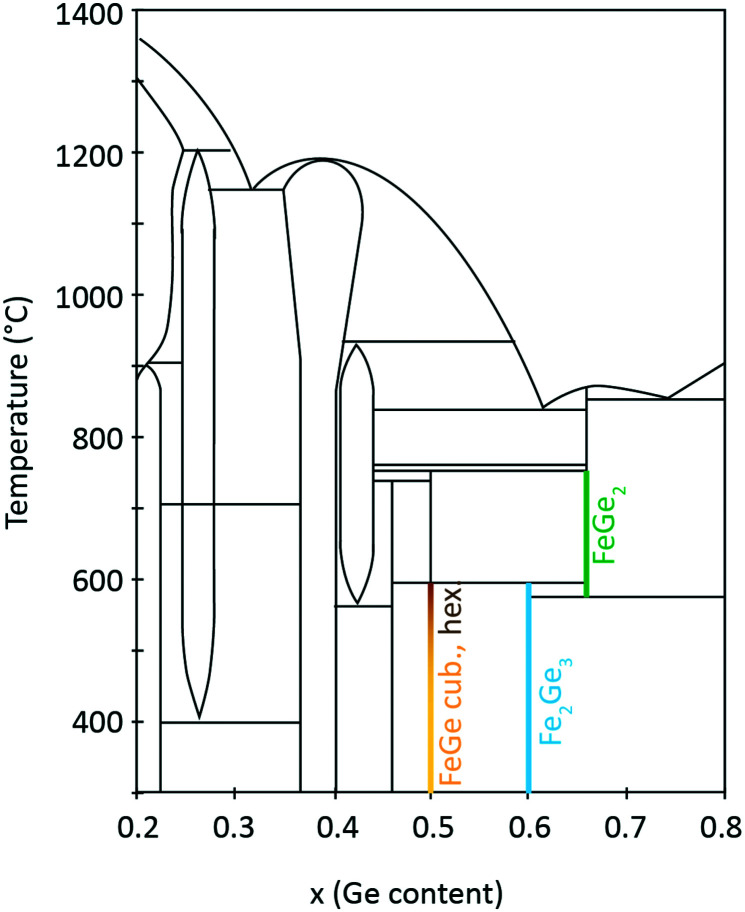
Fe–Ge temperature–composition phase diagram. Adapted from ref. 37.

Fe–Ge exhibits a rich phase diagram. The region for *x* (Ge composition) = 0.2–0.8 is shown in Fig. 1. Many binary compounds between Fe and Ge have been reported, especially in the region from 35 to 50 at% Ge. These include Fe_3_Ge (*P*6_3_/*mmc* (194) and *Fm*3̄*m* (225)),^26^ Fe_2_Ge (*P*6_3_/*mmc* (194)),^26^ Fe_7_Ge_5_ (*P*6_3_/*mmc* (194)),^26^ Fe_1.67_Ge (*P*6_3_/*mmc* (194)),^27^ Fe_5_Ge_3_ (*P*6_3_/*mmc* (194)),^28^ Fe_13_Ge_8_ (*P*6_3_/*mmc* (194)),^26^ Fe_3.2_Ge_2_ (*P*6_3_/*mmc* (194)),^27^ Fe_0.615_Ge_0.385_ (*P*6_3_/*mmc* (194)),^27^ Fe_3_Ge_2_ (*P*6_3_/*mmc* (194)),^26^ Fe_1.4_Ge (*P*6_3_/*mmc* (194)),^27^ Fe_6_Ge_5_ (*C*2/*m* (12)),^26^ FeGe (hexagonal B35) (*P*6/*mmm* (191)),^26^ FeGe (cubic B20) (*P*2_1_3 (198)),^26^ FeGe_2_ (*I*4/*mcm* (140)),^26^ Fe_2_Ge_3_ (*P*4̄*c*2 (116)),^29^ and Fe_90_Ge_10_ (*Im*3̄*m* (229)).^16^

FeGe at a 1 : 1 composition ratio crystallizes in three polymorphs with monoclinic, hexagonal and cubic structures. The cubic B20 structure is stable at temperatures below 580 °C. Above this temperature the hexagonal and monoclinic phases have been identified.^30^ On the Fe-rich side of the phase diagram one finds Fe_6_Ge_5_. On the Ge-rich side, tetragonal Fe_2_Ge_3_ is stable below 600 °C and FeGe_2_ is stable above this temperature. These iron-containing phases exhibit different critical temperatures *T*_c_ for magnetic phase transitions. It was shown in the literature that hexagonal FeGe enters an antiferromagnetic (AFM) phase below a temperature of *T*_c,hex_ = 410 K.^8^ Two magnetic anomalies were observed in hexagonal FeGe single crystals at 30 K and 60 K.^31^ For cubic FeGe, the critical temperature is *T*_c,cub_ = 271 K.^19^ These values should be compared to critical temperatures known for Fe-containing oxides. For magnetite Fe_3_O_4_, a *T*_c_ of 830 K was reported.^32,33^ Further oxides have smaller *T*_c_ such as 743 K in the case of γ-Fe_2_O_3_,^34^ 585 K in the case of ε-Fe_2_O_3_ (ref. 35) and 264 K in the case of hematite α-Fe_2_O_3_.^36^

## 3 Methods

### 3.1 Sample preparation

Fe and Ge multilayer thin films were deposited by magnetron sputtering (Alliance Concept DP-650) at room temperature with a background pressure of 1 × 10^−6^ mbar from 99.999% purity targets. 30 sccm of Ar working gas was introduced prior to sputtering (measured using a mass flow controller) increasing the pressure in the chamber to 5 × 10^−2^ mbar for Fe deposition and 5 × 10^−3^ mbar for Ge deposition. We used an RF source of 300 W and a DC source of 200 W for Fe and Ge, respectively.

**Fig. 2:**
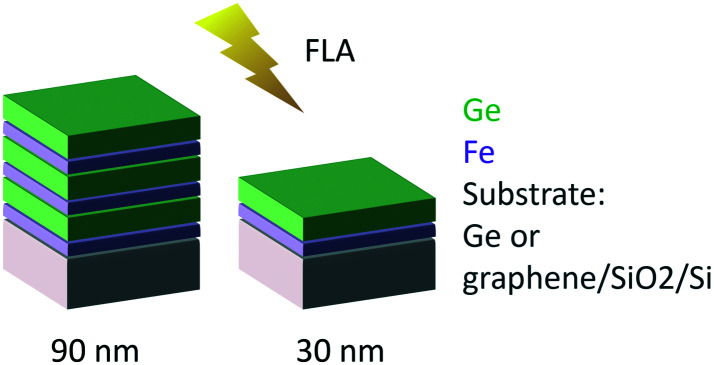
Layer sequences of the annealed samples and schematics of the experiment.

The samples were subsequently crystallized by ultra-fast flash-lamp annealing (FLA). We used an FLA Rovak semi-line system delivering the energy density (pulse length) between 0.3 J cm^−2^ (0.3 ms) and 48 J cm^−2^ (10 ms). We varied the multilayer sequences and individual layer thicknesses (as schematically indicated in Fig. 2) as well as the annealing cycles. Two different nominal total thicknesses of 30 and 90 nm were explored. To vary the heat sink during the FLA process, two different substrates were used: germanium and graphene on oxidized silicon. Table 1 summarizes the sample characteristics and annealing cycles presented here. Prior to the flash annealing, the samples were heated to a pre-heating temperature *T*_pre-heat_ of 200 or 340 °C, as indicated in Table 1, to facilitate the crystallization process.

**Table 1:** Parameters of the FeGe samples: substrate (g – graphene), number of layers *n*_layers_, total nominal thickness *d*, Fe (Ge) layer thickness *d*_Fe_ (*d*_Ge_), nominal atomic ratio Fe/Ge, FLA energy density *E*, pulse length *t* and pre-heating temperature *T*_pre-heat_

Sample	Substrate	*n*_layers_	*d* (nm)	*d*_Fe_ (nm)	*d*_Ge_ (nm)	At. ratio	*E* (J cm^−2^)	*t* (ms)	*T*_pre-heat_
#186	Ge(100)	2	31.2	11.8	19.4	1.2	20	2	340
#192	Ge(100)	6	94.1	10.5	20.9	1	20	2	340
#240	g/SiO_2_/Si(100)	2	31.4	10.5	20.9	1	29	5	200

### 3.2 Lattice dynamics calculations

First principles lattice dynamics calculations based on DFT of FeGe_*x*_ phases with *x* = 1, 1.5, and 2 were performed within the generalized gradient approximation using the modified Perdew–Burke–Ernzerhof local functional PBESol,^37^ as implemented in the CASTEP code.^38^ Norm-conserving pseudopotentials were used. The cut-off energy for the plane wave basis set was set to 800 eV. A self-consistent-field (SCF) tolerance better than 10^−7^ eV per atom and a phonon SCF threshold of 10^−12^ eV per atom were imposed. Prior to performing calculations, the structures were relaxed so that forces on atoms in the equilibrium position did not exceed 3 meV A^−1^ and the residual stress was below 5 × 10^−3^ GPa.

### 3.3 Characterization

TEM and EDX data were obtained using an FEI Tecnai Osiris TEM. We prepared cross-sections with a focused ion beam (FIB) using Ga ions. The sample deposited on graphene was peeled off and directly transferred to a TEM grid (Fig. S1 in the ESI†).

GIXRD patterns were recorded with a Malvern PANalytical Empyrean XRD diffractometer with a monochromatic Cu source at an incidence angle of 5° with a 1D detector. GIWAXS measurements were performed on a D8 Discover Plus TXS (Bruker) with a monochromatic Cu source at incidence angles of 1.0, 2.5 and 4.0°. Bragg–Brentano measurements were performed on a D8 Discover Vario (Bruker) instrument equipped with a Johansson Kα_1_ monochromator.

The Raman spectra were acquired with a confocal Raman spectroscope (Renishaw inVia) using 488 nm excitation. An excitation power density of about 50 W cm^−2^ was utilized to inhibit thermal effects or the damage of the investigated materials.

The magnetization of the films was measured *via* vibrating sample magnetometry (VSM). We recorded the magnetic hysteresis loops *M*(*H*) and the temperature scans using a physical property measurement system (PPMS) from Quantum Design. The samples were cleaved into chips of approximately 4 × 1.5 mm^2^ area. The external field *H* was applied in the plane of the films along the long edge of the chips, *i.e.*, along the easy axis direction attributed to their shape. The hysteresis data were taken in the range of −150 and +150 mT during the zero-field warming (ZFW) at 5, 50, 100, 150, 200, 250, 300 and 400 K. The temperature warming rate was set to 50 K min^−1^. We used a field sweep rate of 0.5 mT s^−1^ (3 mT min^−1^) and the magnetization was averaged during 10 s per measurement. We acquired the temperature scans applying the field cooling (FC) protocol from 400 K to 5 K using different in-plane field values between +90 and −90 mT. The sample was saturated at +200 mT at 400 K before every scan. The temperature sweep rate was 5 K min^−1^.

## 4 Crystal structures and lattice dynamics calculation of the Fe–Ge system

Lattice dynamics calculations of FeGe_*x*_ phases with *x* = 1, 1.5, and 2 (cubic FeGe, hexagonal FeGe, tetragonal FeGe_2_ and tetragonal Fe_2_Ge_3_) were performed.

**Fig. 3:**
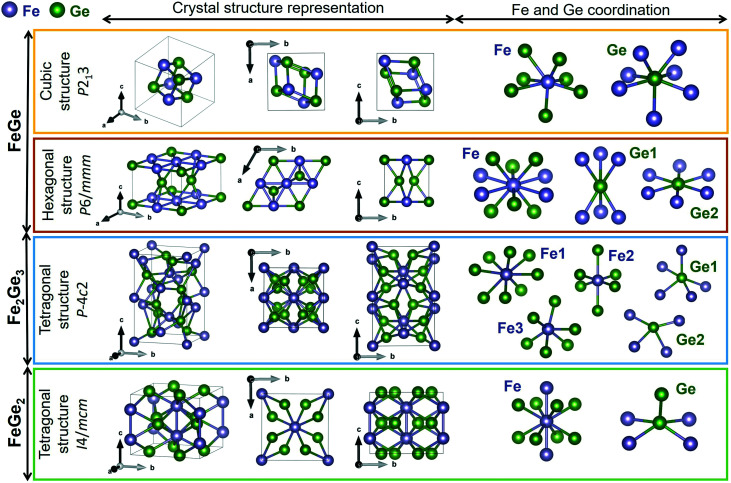
Crystal structure representations of cubic FeGe, hexagonal FeGe, tetragonal Fe_2_Ge_3_ and tetragonal FeGe_2_. Visualized with Vesta^39^ with the crystallographic files from ref. 15 and 41–43.

The optimized crystal lattice parameters were found to be in agreement with the experimentally determined ones.^15,40–42^ The vibrational properties of these structures were further evaluated by the finite displacement method, using an appropriate supercell. Fig. 3 shows the crystal structure representations of cubic FeGe (B20), hexagonal FeGe, tetragonal FeGe_2_ and tetragonal Fe_2_Ge_3_, along with the Fe and Ge coordination in each compound.

Cubic FeGe (B20) crystallizes in the space group 198 or *P*2_1_3 symmetry. The Fe atom is bonded in a 7-coordinate geometry to 7 Ge atoms. There is a variation in the Fe–Ge bond lengths ranging from 2.38–2.62 Å. Ge is bonded in a 7-coordinate geometry to 7 Fe atoms. Hexagonal FeGe crystallizes in the space group 191 or *P*6/*mmm*. The Fe atom is bonded in a 10-coordinate geometry to 4 equivalent Fe and 6 Ge atoms. All Fe–Fe bond lengths are 2.49 Å. There are four shorter (2.48 Å) and two longer (2.49 Å) Fe–Ge bond lengths since there are two inequivalent Ge sites. In the first Ge site, Ge is bonded in a 6-coordinate geometry to 6 Fe atoms. In the second Ge site, Ge is bonded in a hexagonal planar geometry to 6 Fe atoms. Tetragonal Fe_2_Ge_3_ has a Nowotny chimney ladder (NCL) phase with the space group 116 or *P*4̄*c*2. In the first Fe site, Fe is bonded in an 8-coordinate geometry to eight Ge atoms. There are 4 shorter (2.41 Å) and 4 longer (2.63 Å) Fe–Ge bond lengths. In the second Fe site, Fe is bonded in a 6-coordinate geometry to 6 Ge atoms. The Fe–Ge bond distances are between 2.33 and 2.51 Å. In the third Fe site, Fe is bonded in a 6-coordinate geometry to 6 Ge atoms. There are four shorter (2.31 Å) and two longer (2.55 Å) Fe–Ge bond lengths. There are two inequivalent Ge sites. In the first Ge site, Ge is bonded in a 5-coordinate geometry to 5 Fe atoms. In the second Ge site, Ge is bonded in a 4-coordinate geometry to four Fe atoms. Tetragonal FeGe_2_ crystallizes in the space group 140 or *I*4/*mcm*, the prototypical Al_2_Cu structure. Fe is bonded in a 10-coordinate geometry to 2 Fe and 8 Ge atoms. Both Fe–Fe bonds are 2.47 Å long. The Fe–Ge bond lengths are 2.55 Å. Ge is bonded in a 5-coordinate geometry to 4 Fe atoms and one Ge atom. The Ge–Ge bond is 2.63 Å long.

Group theory analysis predicts the following set of Raman active modes based on the irreducible representations of the four Fe–Ge structures at the *Γ* point of the Brillouin zone:FeGe (B20, *P*2_1_3): *Γ* = 2A + 2E + 5T,FeGe (hex, *P*6/*mmm*): *Γ* = E_2g_,Fe_2_Ge_3_ (*P*4̄*c*2): *Γ* = 5A_1_ + 5B_1_ + 7B_2_ + 16E,FeGe_2_ (*I*4/*mcm*): *Γ* = A_1g_ + B_1g_ + B_2g_ + 2E_g_,It should be noted that A and B modes are non-degenerate, E modes are doubly degenerate, and T modes are triply degenerate.

**Fig. 4:**
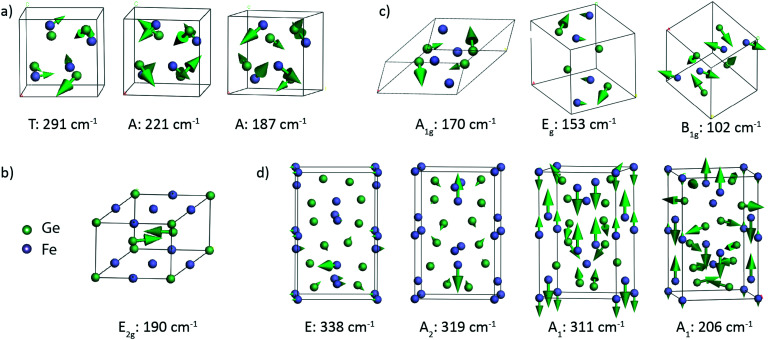
Representative atomic displacements of the calculated vibrational modes corresponding to a) cubic FeGe phonons at 291, 221 and 187 cm^−1^, b) hexagonal FeGe phonon at 190 cm^−1^, c) FeGe_2_ phonons at 170, 153 and 102 cm^−1^ and d) Fe_2_Ge_3_ phonons at 338, 319, 311 and 206 cm^−1^. The arrows indicate the direction of displacement, and their sizes show the relative amplitudes of the atomic motions.

Table 2 presents the theoretically calculated frequencies and symmetry of the Raman active modes expected in these four phases. Atomic displacements of the Raman modes were additionally calculated to provide the visualization of the corresponding atom motions. Fig. 4 shows the representative vibrational patterns of several modes within each Fe–Ge structure. In the case of the cubic FeGe (B20) structure, a total of 9 Raman active modes are expected in the region from 130 to 300 cm^−1^. In most cases the vibrational patterns correspond to the motion of both Ge and Fe, as presented in Fig. 4a. In contrast to this, the hexagonal structure is characterized by only 1 Raman active mode, which corresponds to purely Ge-vibrations expected at 190 cm^−1^ (Fig. 4b). This motion consists of two Ge atoms positioned on the top of two tetrahedra with an Fe base, which symmetrically stretch along the diagonal.

**Table 2:** DFT calculated Raman modes (symmetry and wavenumber) for cubic FeGe, hexagonal FeGe, tetragonal Fe_2_Ge_3_ and tetragonal FeGe_2_

Cubic FeGe (*P*2_1_3)		Hex. FeGe (*P*6/*mmm*)		Fe_2_Ge_3_ (*P*4̄*c*2)		Fe_2_Ge_3_ (*I*4/*mcm*)	
E	133, 242	E_2g_	190	E	57, 89, 114, 126, 167, 168, 182, 186, 210, 232, 253, 270, 281, 288, 327, 338	B_1g_	102
T	142, 191, 227, 252, 291			A_1_	311, 206, 196, 149, 82	B_2g_	122
A	187, 221			B_1_	279, 218, 198, 171, 152	E_g_	153, 167
				B_2_	287, 213, 194, 171, 149, 132, 129	A_1g_	170

The tetragonal FeGe_2_ structure exhibits 5 Raman-active modes expected in the range between 102 and 170 cm^−1^. Fig. 4c shows 3 modes: the B_1g_ mode at 102 cm^−1^, the A_1g_ mode at 170 cm^−1^ and the E_g_ mode at153 cm^−1^. The B_1g_ and E_g_ modes are characterized by combined vibrations of Fe and Ge atoms. The A_1g_ mode at 170 cm^−1^ is purely Ge atom vibrations in the *xz* and *xy* planes.

The tetragonal Fe_2_Ge_3_ phase is characterized with 33 Raman active modes, covering the region from 50 to 340 cm^−1^, whose representative vibrational patterns are given in Fig. 4d. We visualize the E mode at 338 cm^−1^, A_2_ mode at 319 cm^−1^, A_1_ mode at 311 cm^−1^ and A_1_ mode at 206 cm^−1^. These modes come from a combined vibrational motion of Fe and Ge atoms. The atomic displacements are more pronounced for low frequency modes when compared to those for high frequency ones. The lower frequency modes are characterized by in-plane 2D motions, while the high frequency modes are more complex and represented by vibrations in 3D.

## 5 Experimental results and discussion

### 5.1 Film morphology and composition maps

**Fig. 5:**
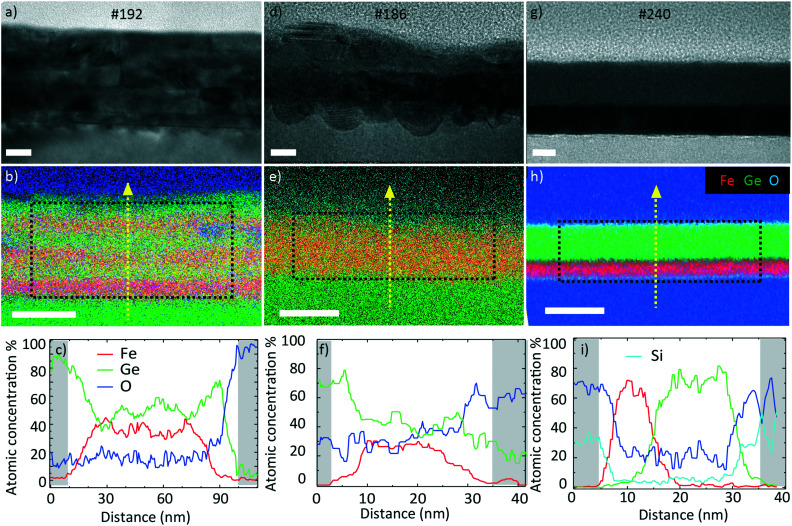
TEM, EDX and composition (from top to bottom) of FeGe samples a–c) #192, d–f) #186 and g–i) #240. Scale bars represent a) 20 nm, d) 10 nm, g) 10 nm, b, e and h) 50 nm. The compositions (line scans in c, f and i) were evaluated along the vertical yellow lines shown in the central row. The uneven interfaces in d) are a consequence of the ion beam etching during the lamella preparation.

We start by presenting the analysis of the crystal structure and composition of the annealed films performed by TEM. Fig. 5 presents the representative TEM micrographs and EDX composition maps as well as the corresponding compositions as line scans.

The data on sample #192 are shown in Fig. 5a–c. Fig. 5a corresponds to the bright field (BF) TEM image of the layer. We observe a slight horizontal contrast, showing the original layered configuration before annealing. The EDX map in Fig. 5b confirms the compositional variation across the sample thickness. The line scan in Fig. 5c provides quantitative information on the variation. The Ge (Fe) content varies between 41 (59) and 64 (36)%. This indicates that the elements mixed during the annealing process. The mixing was not completely homogeneous. The layered nature of the sample was still apparent in the contrast and composition (line scan), as some layers exhibited a higher Ge content than others. The elemental mapping indicated that the surface was oxidized. We did not detect a significant oxygen content in the core of the sample. The average atomic Fe composition in the central region is 47 ± 2%. This was quantified using the Cliff–Lorimer method assuming a thickness of 50 nm and an atomic density of 8 g cm^−3^.

Fig. 5d–f present the data obtained on sample #186. Fig. 5d shows the bright field (BF) TEM image. Similar to the previous sample, we distinguish a slight contrast variation in the growth direction. The EDX map in Fig. 5e shows the Fe–Ge intermixing and oxygen layer. The line scan in Fig. 5f reveals that the Ge (Fe) atomic composition lies between 45 (55) and 78 (22)%. The average atomic Fe composition in the centre is 39 ± 2% which is lower than in the case of the thicker #192 sample with several interfaces.

The data for sample #240 are shown in Fig. 5g–i. The BF TEM image in Fig. 5g shows a clear contrast variation in the growth direction suggesting a lower level of intermixing compared to the two previous samples. The EDX map in Fig. 5h confirms the Fe- and Ge-rich regions. The line scan in Fig. 5i reveals that the layers are intermixed only in a 10 nm thick region. The Ge (Fe) content varies from 0 (90) to 98 (0)%. The average Fe atomic composition in this film is 37 ± 2%. All the average compositions are listed in Table 3. A HRTEM micrograph indicates that the Ge is amorphous and the Fe is nanocrystalline (Fig. S2†), supported by the electron diffraction result (Fig. S1b†).

**Table 3:** Average atomic composition measured by STEM-EDX in the areas depicted by black rectangles in Fig. 5c, f, and i

Sample	#192	#186	#240
Avg. at. Fe content (%)	47 ± 2	39 ± 2	37 ± 2

It should be mentioned that the observed limited intermixing of layers at the back of the samples is due to the short penetration depth of the light during the FLA treatment, resulting in mostly superficial heating.

### 5.2 Phase identification by X-ray diffraction and Raman spectroscopy

Phase identification of the synthesised thin films was performed using Raman spectroscopy and XRD measurements in various configurations, including GIXRD, GIWAXS and the Bragg–Brentano geometry (*θ*–2*θ*). By varying the incidence angles during the XRD measurements, we were able to perform in-depth characterization of the films. Lower values of incidence angles allow investigating the surface region of the thin films, while higher angles correspond more to characterization of the bulk. Finally, the Bragg–Brentano geometry (*θ*–2*θ*) allows characterization of the complete sample, including the thin film and substrate layers. The experimental XRD data were compared with the calculated XRD patterns obtained from the Crystallography Open Database.^43^

**Fig. 6:**
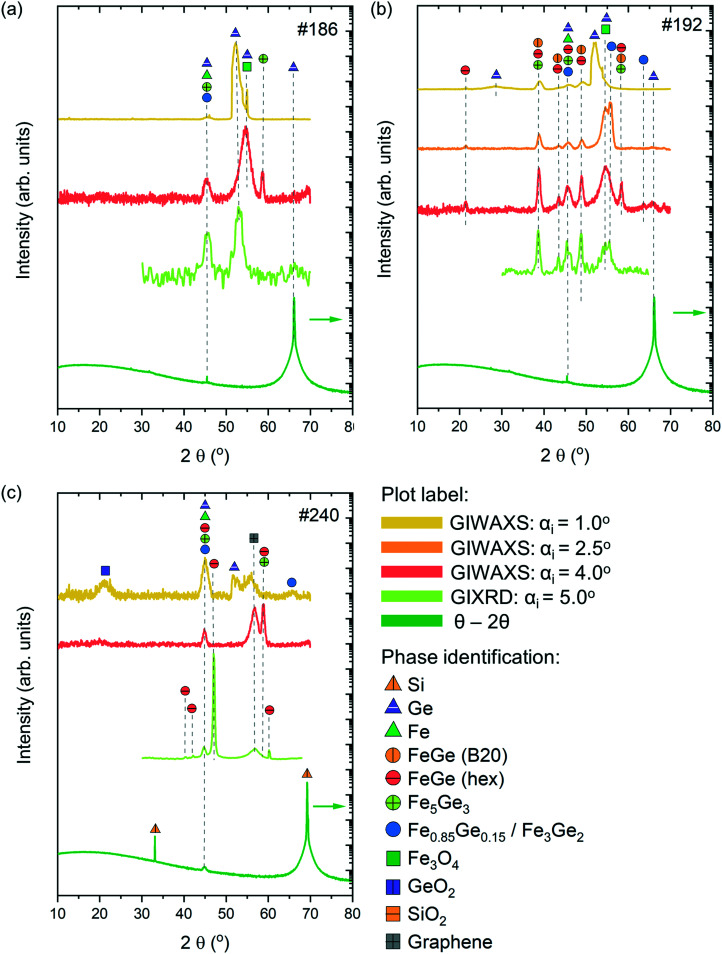
XRD patterns of (a) #186, (b) #192 and (c) #240 samples measured in GIWAXS (incidence angles *α*_i_ = 1.0, 2.5 and 4°), GIXRD (incidence angle *α*_i_ = 5.0°) and Bragg–Brentano (*θ*–2*θ*) configurations, along with the identification of peaks corresponding to different phases. Note that the patterns corresponding to the Bragg–Brentano configuration are presented on the logarithmic scale (pointed with the arrow).

Fig. 6 presents the XRD patterns from the three investigated samples which were measured in various configurations, along with the phase identification.

Sample #186 exhibits a relatively simple XRD pattern with the appearance of 2–3 peaks depending on the measuring configuration. According to the GIWAXS measurements with *α*_i_ = 1.0 and 4°, the surface and subsurface regions of the sample are characterized by the presence of Fe-rich phases, Fe_3_Ge_5_ and Fe_0.85_Ge_0.15_/Fe_3_Ge_2_ phases. Note that the XRD patterns of Fe_0.85_Ge_0.15_ and Fe_3_Ge_2_ are very similar, making it difficult to differentiate at the level of our measurements. Besides these phases, contributions from Fe_3_O_4_ and unreacted Ge and Fe have also been observed. GIXRD measurements with *α*_i_ = 5.0° and Bragg–Brentano measurements reveal mostly the presence of Fe_0.85_Ge_0.15_/Fe_3_Ge_2_ in the bulk of the film, along with the unreacted Fe and Ge contributions from the substrate.

Sample #192 presents a more complex pattern with a higher number of contributions. The surface of the sample corresponding to GIWAXS measurements with *α*_i_ = 1.0 and 2.5° is mostly dominated by unreacted Ge and Fe_3_O_4_. Iron rich Fe_0.85_Ge_0.15_/Fe_3_Ge_2_ is the major phase present in this region, with smaller contributions of cubic FeGe (B20) and hexagonal FeGe. These last two phases become dominant in the bulk of the sample as observed with higher intensity peaks corresponding to FeGe (B20) and hexagonal FeGe phases in patterns obtained from GIWAXS and GIXRD measurements with *α*_i_ = 4.0 and 5.0°, respectively. A small contribution of unreacted Fe to the reflection at 2*θ* = 45° is expected for all grazing incidence measurements. The Bragg–Brentano measurements are mostly characterized with Ge peaks belonging to the substrate.

GIWAXS measurements with *α*_i_ = 1.0° show that the surface region of sample #240 is characterized by the presence of Fe_0.85_Ge_0.15_/Fe_3_Ge_2_ along with contributions from unreacted Fe and Ge, along with GeO_2_ and graphene from the substrate. Fe_3_Ge5 is the dominant phase in the subsurface region corresponding to GIWAXS measurements with *α*_i_ = 4.0°, while the presence of the hexagonal FeGe phase is observed in the bulk of the film (GIXRD measurements with *α*_i_ = 5.0°). The Bragg–Brentano measurements reveal mostly contributions from Si belonging to the substrate. The GIXRD measurements of the substrate can be found in the ESI† (Fig. S4).

**Fig. 7:**
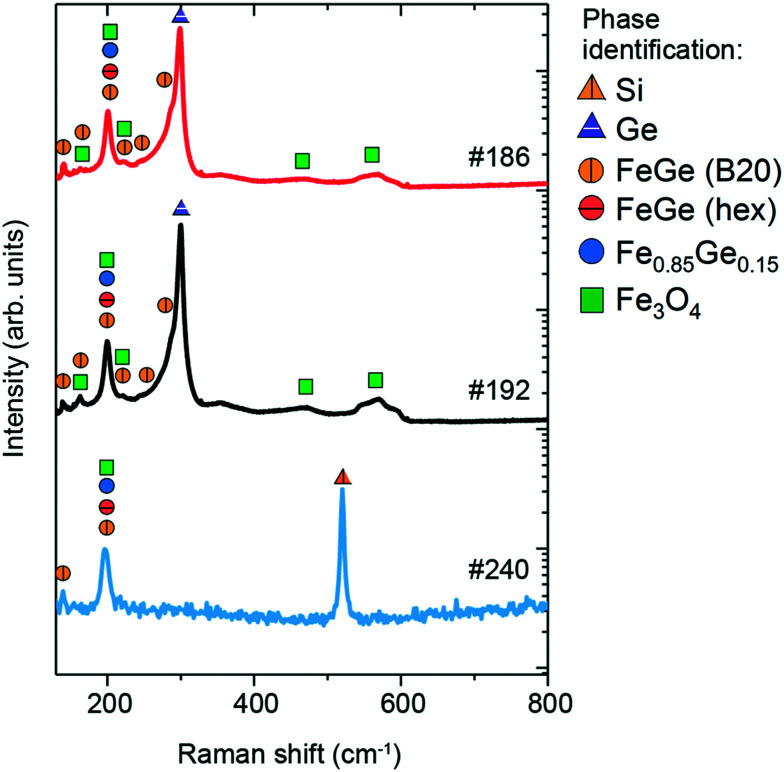
Raman spectra of #186, #192 and #240 thin films and the comparison with the reference values.

The Raman spectra of the three samples are shown in Fig. 7, along with phase identification of peaks (enlarged features of the low-intensity peaks are presented in Fig. S5 in the ESI†). Sample #186 exhibits 9 features in the measured region of 100–600 cm^−1^. The most prominent peaks are located at 200 and 300 cm^−1^, while the other low-intensity peaks are at 139, 163, 221, 244, 290, 467 and 560 cm^−1^. The peak at 300 cm^−1^ is typical of Ge-vibrations^44^ and can be assigned to unreacted Ge that is observed in the surface region by XRD and EDX measurements or from the substrate. The other dominant peak at 200 cm^−1^ is probably due to the Fe-rich Fe_0.85_Ge_0.15_ phase according to Wang *et al.*^16^ who have observed a similar Raman contribution in their Fe–Ge nanoparticles. This peak can be also assigned to hexagonal FeGe and cubic FeGe (B20) phases. Hexagonal FeGe and FeGe (B20) have not been observed in the XRD measurements for this sample, in contrast to the Fe_0.85_Ge_0.15_ phase. However, this does not exclude their presence, as Raman spectroscopy is a microscopic technique which can detect nanometric traces of materials, in contrast to XRD which is macroscopic and requires a significant amount of material in order to show the presence in the patterns. Furthermore, the lower intensity peaks at 139, 163, 221, 244 and 290 cm^−1^ agree quite well with the expected Raman peaks for the cubic FeGe (B20) phase based on the calculations, as shown in Table 2. The contribution from the Fe_3_O_4_ phase is seen in the presence of the Raman peaks at 467 and 560 cm^−1^, as well as minor contributions at 163, 200 and 221 cm^−1^, all of which agree with the XRD results.

The Raman spectrum of sample #192 also contains 9 features. Similar to sample #186, the most prominent features are located at 201 and 299 cm^−1^, while lower intensity peaks are located at 141, 162, 222, 244, 288, 467 and 560 cm^−1^. As discussed previously, the peak at 300 cm^−1^ is assigned to unreacted Ge observed in the surface of the sample from XRD and EDX measurements or from the substrate. The peak at 201 cm^−1^ is due to Fe_0.85_Ge_0.15_, hexagonal FeGe and FeGe (B20), all of which have been observed in the XRD patterns. Lower intensity peaks at 141, 162, 222, 244 and 288 cm^−1^ are typical of the cubic FeGe (B20) phase. The peaks at 467 and 560 cm^−1^, as well as minor contributions at 162, 201, and 222 cm^−1^, are attributed to Fe_3_O_4_, whose presence is additionally confirmed by XRD measurements.

The Raman spectrum of sample #240 exhibits 3 peaks at 138, 201 and 519 cm^−1^. The peak at 519 cm^−1^ is assigned to the Si substrate, while the contribution at 201 cm^−1^ is due to the presence of Fe_0.85_Ge_0.15_ and hexagonal FeGe, both of which have been observed in XRD measurements. The peak at 138 cm^−1^, along with a small contribution at 201 cm^−1^, indicates the presence of the nanometric FeGe (B20) phase, which was not detected in the XRD measurements. It should be noted that while GeO_2_ has been observed in the XRD measurements, no clear contributions are seen in the Raman spectrum of this sample.

Table 4 summarises the peak positions observed in the Raman spectra of the three samples, as well as their assignment to different phases.

**Table 4:** Summary of observed peaks in the Raman spectra of #186, #240, and #192 samples and the phase assignment according to DFT calculations (cubic and hexagonal FeGe) in this work and the literature (Ge,^44^ Si, Fe_3_O_4_ (ref. 45) and GeO_2_,^46^ Fe_0.85_Ge_0.15_ (ref. 16))

Experimental (wavenumber)			Identified phases	Calculated (this work) (wavenumber, symmetry)				Literature (wavenumber)				
#186	#240	#192		Cubic FeGe		Hex. FeGe		Ge	Si	Fe_3_O_4_	GeO_2_	Fe_0.85_Ge_0.15_
139	138	141	Cubic FeGe	142	T						124	
163		162	Cubic FeGe, Fe_3_O_4_	187	A					160	168, 181	
200	201	201	Cubic FeGe, hex. FeGe, Fe_0.85_Ge_0.15_, Fe_3_O_4_	191	T	190	E_2g_			193		200
221		222	Cubic FeGe, Fe_3_O_4_	221, 227	A, T					226		
244		244	Cubic FeGe	242, 252	E, T							
290		288	Cubic FeGe	291	T							
300		299	Ge					300			302	
467		467	Fe_3_O_4_							470	444	
	519		Si						519			
560		560	Fe_3_O_4_							560		

### 5.3 Magnetic properties

**Fig. 8:**
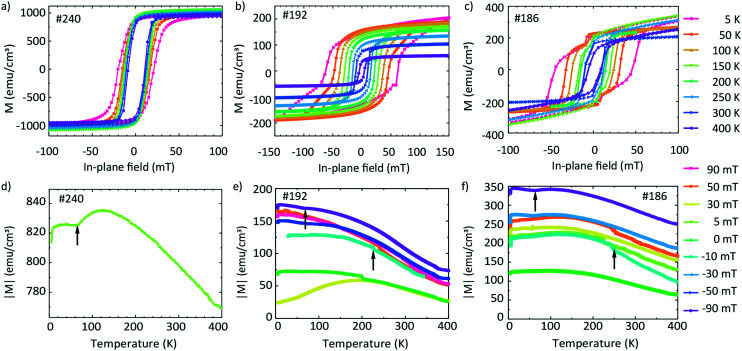
Magnetic hysteresis curves (a–c) and field-cooling temperature scans (d–f) of samples #240 (left), #192 (center), and #186 (right) in in-plane magnetic fields.

Magnetization data are shown in Fig. 8. The top graphs in Fig. 8a–c show the hysteresis loops *M*(*H*) obtained at different temperatures *T* and the bottom graphs in Fig. 8d–f show the temperature-dependent magnetization *M* measured at different fixed field values. The values are expressed in emu cm^−3^ units, where for the volume, we considered the area of the film multiplied by the thickness from the EDX line scans containing Fe.

It is instructive to first discuss the magnetic properties of sample #240 for which Fe and Ge intermixed in a narrow transition region only (Fig. 5i). Fig. 8a shows the hysteresis loops of the sample. The saturation magnetization *M*_s_ does not vary significantly with the temperature. We extract a value of 995 emu cm^−3^ at 300 K and 998 emu cm^−3^ at 5 K. The coercive field *H*_c_(*T*) varies slightly with the temperature. *μ*_0_*H*_c_ increases from 11.2 mT (300 K) to 20.6 mT (5 K). The nearly temperature-independent hysteresis loops in Fig. 8a are consistent with a ferromagnet exhibiting a critical temperature above room temperature such as Fe. We attribute the detected magnetic behavior hence mainly to Fe which did not intermix with Ge. Fig. 8b shows the hysteresis loops of sample #192. We observe that *M*_s_ increases with decreasing temperature from 93 (300 K) to 200 emu cm^−3^ (5 K). The low-temperature value is smaller by a factor of 5 compared to that in Fig. 8a, consistent with better intermixing of Fe with Ge. For *H*_c_(*T*) we obtain *μ*_0_*H*_c_ = 10.1 mT at 300 K and 62.2 mT at 5 K. Below 200 K, the recorded hysteresis loops show a change in the slope below (after) the coercive fields. For 150 K the change in the slope occurs at 40 mT. For higher *T*, this feature is not observed.

The hysteresis loops of sample #186 are shown in Fig. 8c. *M*_s_ increases from the 190 emu cm^−3^ (300 K) to 260 emu cm^−3^ (5 K). We attribute the slight paramagnetic (PM) behaviour to the used sample holder. We observe an increase of *μ*_0_*H*_c_ with decreasing temperature from 9.7 mT (300 K) to 46.8 mT (5 K). The hysteresis loops obtained below 200 K exhibit changes in the slope slightly above (before) the relevant coercive field. These features occur at around 10 mT for loops taken at *T* = 5, 50 and 100 K, *i.e.*, at smaller fields compared to sample #192.

The temperature dependence of the absolute value of magnetization *M*(*T*) measured at 5 mT on sample #240 is shown in Fig. 8d. The absolute value of *M* changes slightly from 840 to 770 emu cm^−3^ in the temperature region from 5 to 400 K.

The absolute values of magnetization *M*(*T*) of sample #192 are shown in Fig. 8e. The graph contains the curves measured during the field-cooled (FC) protocol at 90, 50, 30, 0, −30, −50 and −90 mT. Depending on the field value, the curves are shifted to different absolute values. For +90 and −90 mT, *M*(*T*) decreases by a factor of about two between 5 K and 400 K, *i.e.*, the magnetization varies with *T* much more significantly than that of sample #240 (Fig. 8d) for which Fe and Ge layers were well separated. We measure a non-zero magnetization at 400 K suggesting that the *T*_c_ of the film is above this temperature. We identify two kinks in the *M*(*T*) curves (marked with upward arrows), one near 230 K and one at 70 K. We note here that a low temperature kink is seen also in Fig. 8d.

Fig. 8f shows the *M*(*T*) curves of sample #186 obtained by means of the FC protocol at different fields. Their temperature dependencies and the appearance of kinks are similar to those in Fig. 8e. Still at low temperature the values of *M* are larger by a factor of up to two compared to those of sample #192. The *T*_c_ of the film is also above the measured region of 400 K. The obtained values are summarized in Table 5.

**Table 5:** Magnetic properties of FeGe films of samples #192, #186 and #240 extracted from the hysteresis and temperature scans in Fig. 8. Saturation magnetization measured at 300 K and 5 K, and coercive field at 300 K and 5 K after applying the in-plane field

Sample	*M*_sat_				*H*_c_	
	(emu cm^−3^)		(mT)		(mT)	
	*T* = 300 K	*T* = 5 K	*T* = 300 K	*T* = 5 K	*T* = 300 K	*T* = 5 K
#192	93 ± 4	200 ± 9	117 ± 5	246 ± 11	10.1 ± 0.5	62 ± 3
#186	190 ± 9	260 ± 12	239 ± 11	325 ± 15	9.7 ± 0.4	47 ± 2
#240	995 ± 45	998 ± 45	125 ± 6	125 ± 6	11.2 ± 0.5	20.6 ± 0.9

**Fig. 9:**
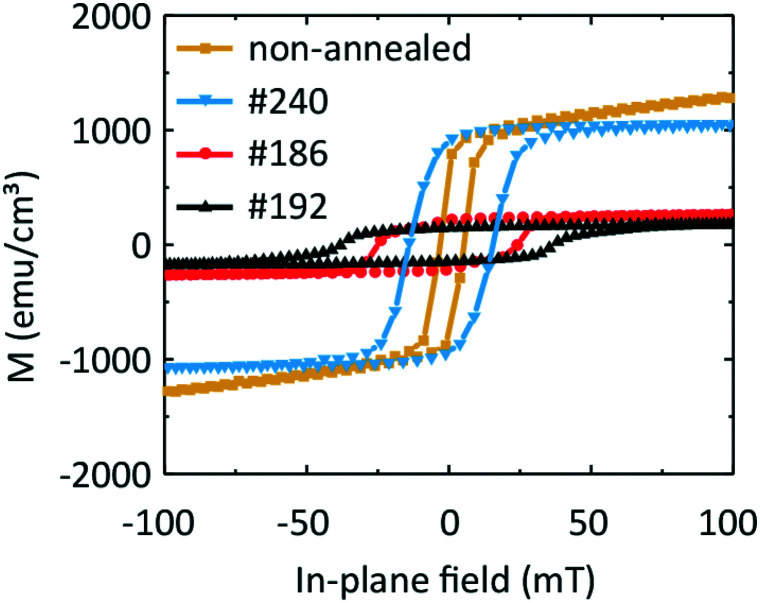
Comparison of the hysteresis loops for samples #240, #186 and #192 with a non-annealed sample at *T* = 100 K. The non-annealed sample contain a nanocrystalline Fe (11.8 nm) layer and an amorphous Ge (19.4 nm) layer.

In Fig. 9 we compare the hysteresis loops obtained on the three samples #240, #192, and #186 at one and the same temperature of 100 K with that of a sample containing an Fe and a Ge layer which was not annealed. The non-annealed sample exhibits an *M*_sat_ value of 1040 emu cm^−3^ (after subtraction of the paramagnetic background). The coercive field *μ*_0_*H*_c_ amounts to 4 mT. Given that the non-annealed sample is composed of sputtered Fe and Ge on a Ge substrate, we would expect the magnetic behaviour of the sample to be similar to that of pure Fe. Considering that the difference in *M*_sat,Fe_ ≈ 1630 emu cm^−3^ in comparison to 1040 emu cm^−3^ measured here, we conclude that the non-annealed sample contains both Fe and F_3_O_4_. We observe similar saturation magnetization values of the non-annealed and #240 samples. This indicates a similarity between the samples' morphology and the lack of pronounced intermixing of Fe and Ge.

Samples #186 and #192 exhibit a change in the slope in their hysteresis loops at low temperatures. We attribute this behaviour to the appearance of a second low-temperature magnetic phase which coexists with the high-temperature magnetic phase detected at around room temperature. This hypothesis is in agreement with the presence of three Fe–Ge crystal phases, the Fe_0.85_Ge_0.15_ and the hexagonal and cubic FeGe. If we compare the coercive field values *μ*_0_*H*_c_ of the non-annealed sample and of sample #240 with that of sample #186, we suggest that the high-temperature phase can be attributed to the phase present in the non-annealed sample, *i.e.*, oxidized Fe.

For the two other samples, we observe two kinks in *M*(*T*) near 230 K and 60 K. These values are close to the critical temperature of cubic FeGe and a magnetic anomaly in hexagonal FeGe, respectively. These observations support the mixture of hexagonal and cubic FeGe phases in annealed samples consistent with the structural and Raman spectroscopy investigations. The low-temperature kink in sample #240 might reflect the intermixing of Fe and Ge in the 10 nm wide transition region identified in Fig. 5. The non-zero magnetization at 400 K can be attributed to the presence of an Fe-rich compound or Fe-containing oxides. The compound Fe_3.34_Ge_2_ exhibits *e.g.* a high critical temperature *T*_c_ of 470 K.^47^ The measured differences between the magnetic characteristics of samples #192 and #186 on the one hand and #240 and a non-annealed sample on the other hand indicate the successful application of FLA leading to Fe–Ge intermixing and crystallization of FeGe phases.

## 6 Conclusions

A series of layered FeGe_*x*_ thin films, subjected to flash-lamp annealing, which was used to intentionally induce intermixing and crystallization of FeGe phases, were studied experimentally by Raman spectroscopy and X-ray diffraction, and the results were substantiated by magnetic measurements. First principles lattice dynamics calculations of Raman modes for cubic and hexagonal FeGe, as well as tetragonal Fe_2_Ge_3_ and FeGe_2_, have been performed and compared with the experimental data. It is shown that depending on the layer design the films contain primary cubic and hexagonal FeGe polymorphs along with Fe-rich phases and pure Ge and Fe. One way to improve the formation of high-quality crystal phases might be by co-sputtering of Fe and Ge, leading to a better intermixing of materials before the FLA treatment. The performed vibrational mode assignments for four distinct FeGe_*x*_ compounds open up a way for fast and local phase determination in thin FeGe films by means of Raman spectroscopy measurements.

## Author contributions

A. F. M. and D. G. conceived the research. A. K. prepared the samples. S. P. R., H. M. and M. B. did the flash-lamp annealing. A. K. did the Raman, GIXRD, TEM, STEM-EDX and magnetization measurements and data analysis with inputs from M. D., A. F. M., and D. G. M. D. did the GIWAXS experiments and data analysis. A. P. L. did the DFT calculations. N. T. did the FLA temperature profile simulation. M. D. and A. K. performed the data interpretation, coordinated the simulations, and connected all results. A. K., M. D., A. F. M. and D. G. wrote the manuscript with inputs from all the authors.

## Conflicts of interest

There are no conflicts to declare.

## Supplementary Material

CE-023-D1CE00970B-s001
