# Supplementary material for: Cubic, hexagonal and tetragonal FeGex phases (x = 1, 1.5, 2): Raman spectroscopy and magnetic properties
Source: CrystEngComm. 2021 Aug 20;23(37):6506–17. doi: 10.1039/d1ce00970b (PMC8474057; doi:10.1039/d1ce00970b)
Supplement: CE-023-D1CE00970B-s001 [file CE-023-D1CE00970B-s001.pdf]

Electronic Supplementary Material (ESI) for CrystEngComm.
This journal is © The Royal Society of Chemistry 2021

Supporting Information for:

Cubic, hexagonal and tetragonal FeGe$_x$ phases (x = 1, 1.5, 2): Raman spectroscopy and magnetic properties

A. Kúkoľová, [‡a,b] M. Dimitrievska, [‡a*] A. P. Litvinchuk, [c] S. P. Ramanandan, [a] N. Tappy, [a] H. Menon, [d,e] M. Borg, [d,e] D. Grundler, [b,f] and A. Fontcuberta i Morral[a,g*]

‡ These authors contributed equally to this work.

a - Laboratory of Semiconductor Materials, Institute of Materials, School of Engineering, Ecole Polytechnique Fédérale de Lausanne (EPFL), 1015 Lausanne, Switzerland.

b - Laboratory of Nanoscale Magnetic Materials and Magnonics, Institute of Materials, Ecole Polytechnique Fédérale de Lausanne (EPFL), 1015 Lausanne, Switzerland.

c - Texas Center for Superconductivity at UH, Department of Physics, University of Houston, USA.

d - Electrical and Information Technology, Lund University, Lund, Sweden.

e - NanoLund, Lund University, Lund, Sweden.

f - Institute of Electrical and Micro Engineering, School of Engineering, Ecole Polytechnique Fédérale de Lausanne (EPFL), 1015 Lausanne, Switzerland.

g - Institute of Physics, School of Basic Sciences, Ecole Polytechnique Fédérale de Lausanne (EPFL), 1015 Lausanne, Switzerland.

*mirjana.dimitrievska@epfl.ch, anna.fontcuberta-morral@epfl.ch

a) b) c)

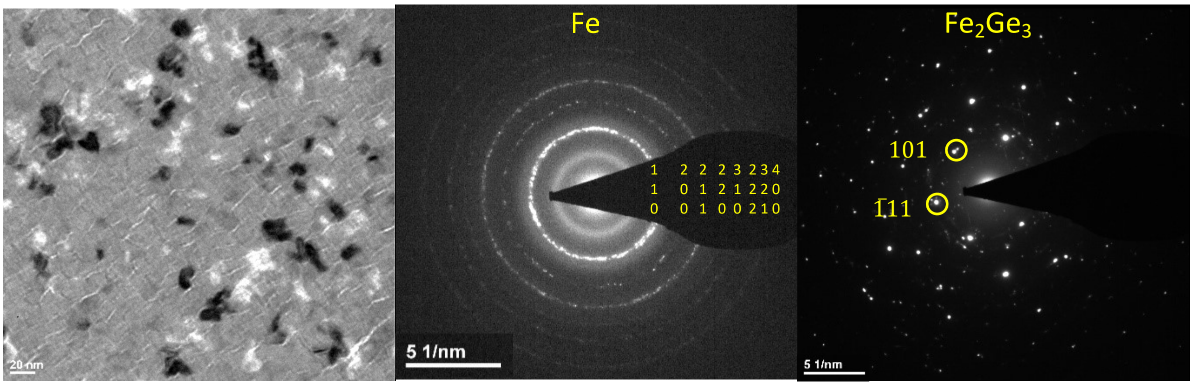

Figure S1: a-b) TEM data on FeGe sample #240 prepared by peeling of the film from the graphene substrate. The bright field TEM top view in a) and the SAED ring pattern matching the Fe phase in b). c) SAED pattern of FeGe sample #94 on graphene annealed at 20 J/cm^2 with the 400°C pre-heating. The pattern match the tetragonal Fe_2Ge_3 phase.

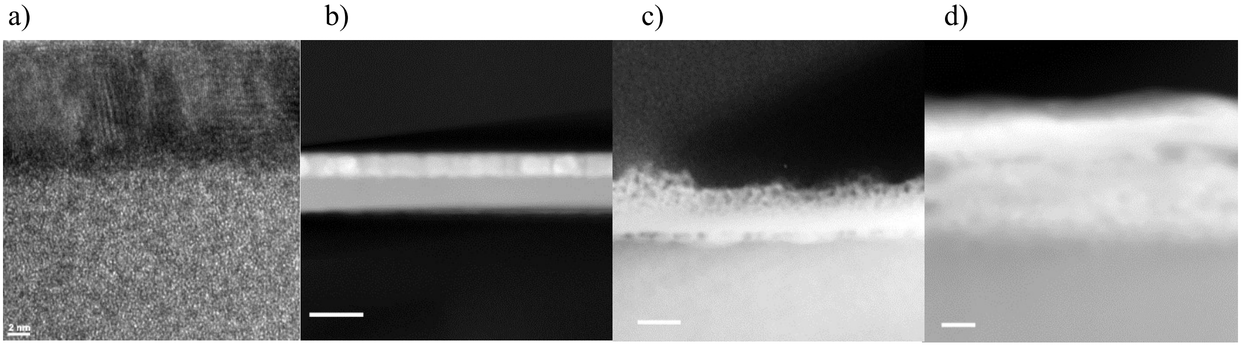

Figure S2: a) HRTEM of cross-section from sample #240 with the nanocrystalline Fe layer on the top, and amorphous Ge layer at the bottom. HAADF micrographs of sample b) #240, c) #186 and d) #192. Scale bar in c-d) 20 nm.

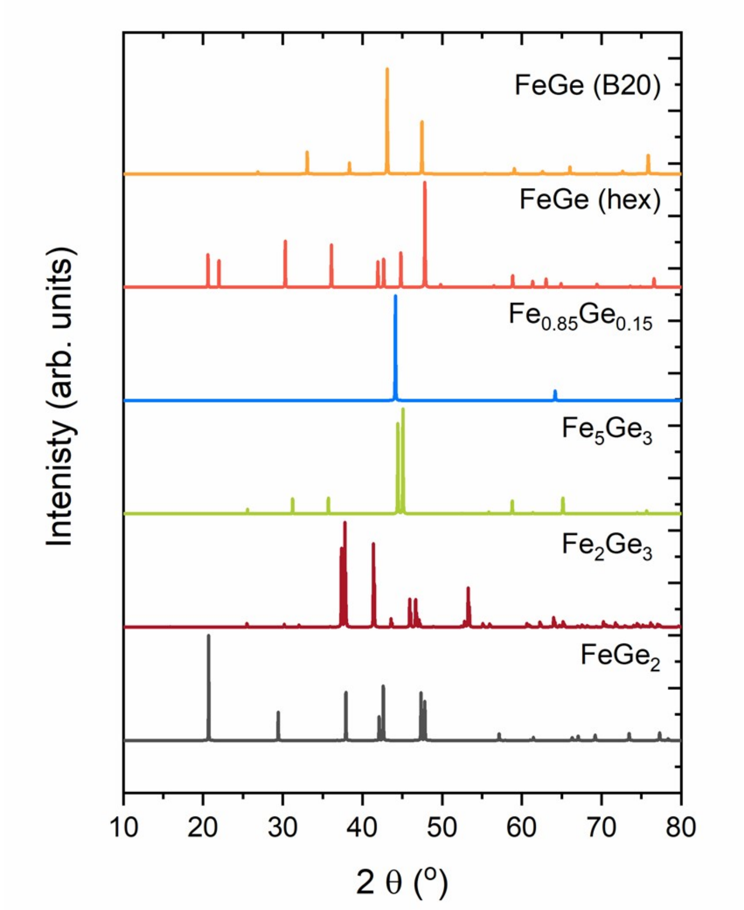

Figure S3: Simulated XRD patterns for cubic FeGe (B20), hexagonal FeGe, $Fe_{0.85}Ge_{0.15}$, Fe_5Ge_3, Fe_2Ge_3, $FeGe_2$.

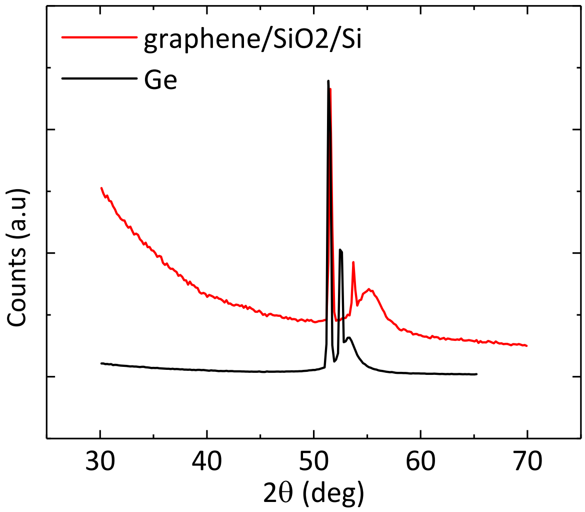

Figure S4: GIXRD of the graphene/SiO2/Si and Ge substrate.

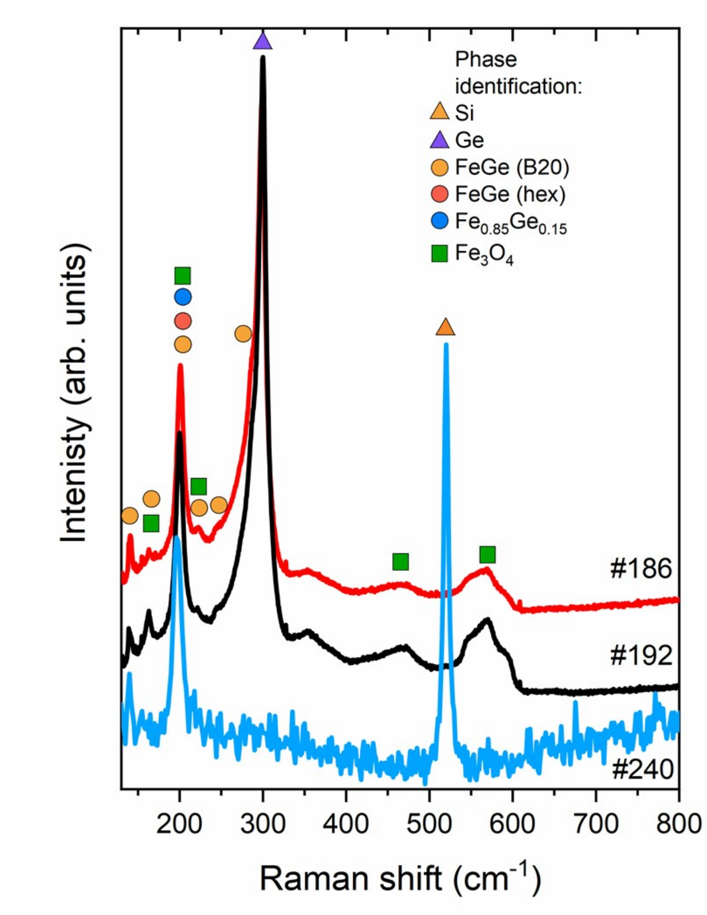

Figure S5. Raman spectra of #186, #192 and #240 thin films and the comparison with the reference values.
